# Association between cardiometabolic Index and obstructive sleep apnea and the mediating role of smoking: a cross-sectional study

**DOI:** 10.3389/fendo.2025.1609585

**Published:** 2025-07-09

**Authors:** Yifan Zhou, Jinghao Yu, Haitao Tan, Jun Xiao

**Affiliations:** ^1^ Department of Orthopedics, The 921st Hospital of the People’s Liberation Army, The Second Affiliated Hospital of Hunan Normal University, Changsha, China; ^2^ Hangzhou Dianzi University, Hangzhou, Zhejiang, China; ^3^ The No. 924 Hospital of the Joint Logistic Support Force of the Chinese People’s Liberation Army, Guilin, China

**Keywords:** CMI, OSA, NHANES, smoking, mediating effect

## Abstract

**Background:**

The cardiometabolic Index (CMI) serves as a metric for evaluating the functional and metabolic health of the heart. It aids healthcare professionals in assessing cardiac health, predicting the risk of cardiovascular diseases, and determining the effectiveness of various treatments. Despite its significance, there is a scarcity of studies examining the relationship between CMI and obstructive sleep apnea (OSA). Consequently, our objective was to clarify the relationship between CMI and OSA.

**Methods:**

We conducted a cross-sectional study using data from the 2015–2018 National Health and Nutrition Examination Survey (NHANES), focusing on a cohort of adults aged 20 years and older. To assess the prevalence of OSA, we employed the Sleep Questionnaire (SLQ) included in the NHANES dataset, which identifies OSA based on symptom-based survey items. Various analytical methods were utilized to examine the relationship between CMI and OSA, including multivariate logistic regression, restricted cubic splines (RCS), threshold effect analysis, subgroup analyses, and mediation effect analyses.

**Results:**

In this study, we included 3,912 participants, among whom 1,997 were diagnosed with OSA, resulting in a prevalence of 51%. After thoroughly accounting for relevant covariates, a positive correlation between the CMI and OSA was observed [OR (95% CI): 1.31 (1.21, 1.42), p < 0.001]. This association was further corroborated through restricted cubic spline (RCS) analyses. Additionally, threshold effect analyses indicated a significant inflection point, with the prevalence of OSA increasing significantly with CMI and then leveling off. Further subgroup analyses demonstrated a significant interaction based on smoking status (p < 0.05). Finally, mediation analyses confirmed that smoking served as a mediator in the relationship between CMI and OSA, exhibiting a mediation effect size of 0.002115.

**Conclusion:**

In the adult population of the United States, a positive nonlinear relationship exists between the CMI and the prevalence of OSA. Smoking status partially mediates this association. Additionally, the findings from the threshold effects analysis indicate that maintaining CMI within an appropriate range can significantly decrease the likelihood of developing OSA.

## 1 Introduction

Obstructive sleep apnea (OSA) is a prevalent sleep disorder marked by recurring instances of partial or complete blockage of the upper airway during sleep, which leads to interruptions in sleep and episodes of intermittent hypoxia (1, 2). This condition impacts a considerable segment of the adult population, with estimates indicating that more than 25% of adults may experience mild to moderate OSA (3, 4). OSA is linked to numerous serious health issues, including an elevated risk of cardiovascular disease, hypertension, type 2 diabetes, and metabolic syndrome (5–8). The chronic nature of OSA, along with the likelihood of many cases remaining undiagnosed (9), emphasizes the necessity for early identification and intervention to reduce its detrimental effects.

In clinical research and practice, composite indices have emerged as valuable tools for capturing complex physiological phenomena through integrated metrics. These indices combine multiple biomarkers or clinical parameters into a single quantitative measure, offering a more holistic assessment than individual variables alone. Particularly in endocrinology and metabolic medicine, composite indices like HOMA-IR for insulin resistance or TyG (triglyceride glucose) index have proven instrumental in evaluating multifaceted conditions (10, 11). Their strength lies in synthesizing interrelated biological processes, enhancing predictive capacity, and simplifying clinical decision-making. However, such indices also present limitations: they may obscure the relative contribution of individual components, vary in performance across populations, and sometimes lack standardized cutoff values. Despite these challenges, when properly validated, composite indices remain powerful tools for uncovering systemic relationships in metabolic health.

The Cardiometabolic Index (CMI) serves as a critical tool for assessing both cardiac function and metabolic status (12, 13). By integrating various parameters—such as body weight, waist circumference, and biochemical markers (14), CMI provides a comprehensive evaluation of an individual’s cardiovascular risk, thus allowing healthcare providers to identify at-risk individuals and implement timely preventive strategies (15, 16). Understanding the significance of CMI is crucial not just within clinical practice but also in research settings, as it has the potential to uncover novel associations between metabolic health and sleep disorders.

Recent studies have begun to explore the complex relationship between CMI and OSA, revealing that elevated CMI may predispose individuals to OSA. Factors such as increased adiposity can exacerbate upper airway obstruction, while systemic inflammation frequently seen in metabolic dysregulation may also play a pivotal role (17–20). Conversely, the metabolic changes associated with OSA, including insulin resistance and dyslipidemia, can further heighten the risks posed by a high CMI, suggesting a bidirectional interplay between the two conditions (21–23). The interplay between CMI and OSA is complex and multifactorial, involving several biological and behavioral mechanisms that warrant further exploration. Therefore, understanding the nuanced connection between CMI and OSA is not merely an academic exercise; it has far-reaching implications for public health strategies. A clearer understanding of how CMI and OSA interact could pave the way for targeted interventions that address both metabolic health and sleep quality, ultimately aiming to reduce the prevalence and impact of OSA in individuals with elevated CMI.

This study aims to elucidate the association between CMI and OSA, contributing to a more comprehensive understanding of their interconnected roles in cardiovascular and metabolic health. We hypothesize that elevated CMI values will demonstrate a positive association with OSA prevalence. Furthermore, investigating potential threshold effects in this relationship represents a critical research objective. Additionally, we aim to examine whether specific mediating factors influence the CMI-OSA association.

## 2 Methods

### 2.1 Study population

The National Health and Nutrition Examination Survey (NHANES) is an extensive cross-sectional study carried out by the National Center for Health Statistics (NCHS), which is a component of the Centers for Disease Control and Prevention (CDC) in the United States. Established in the early 1960s, NHANES seeks to evaluate the health and nutritional status of the U.S. population by utilizing a combination of interviews, physical examinations, and laboratory tests. Data in the NHANES database is collected from a representative sample of civilians, covering various demographic groups across the country. The survey employs a multistage sampling design to ensure that the findings are generalizable to the broader U.S. population.

**Figure 1:**
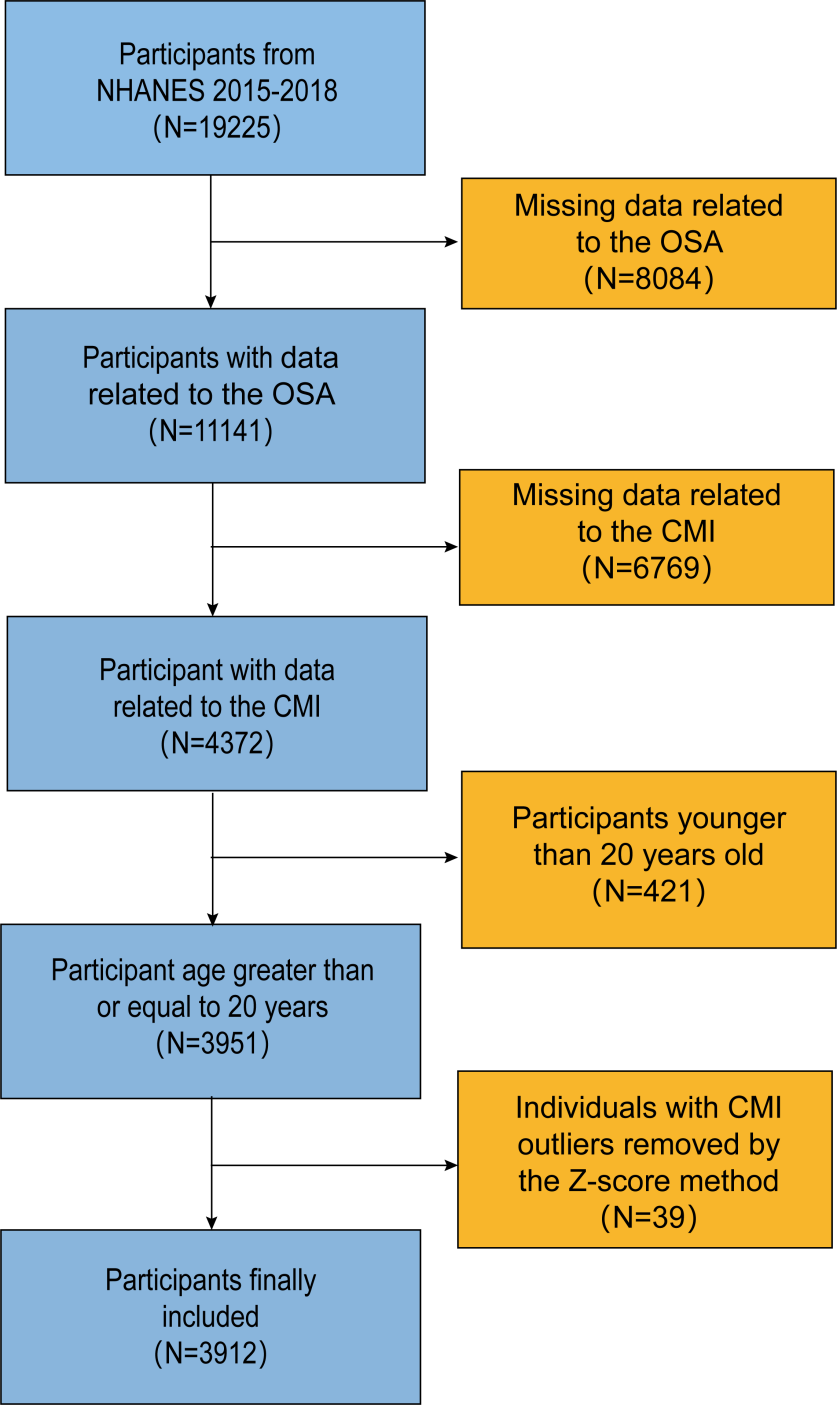
Inclusion exclusion flowchart.

We utilized data from two cycles of the National Health and Nutrition Examination Survey (NHANES) conducted between 2015 and 2018. The initial dataset included 19,225 participants. However, after excluding cases with incomplete information on OSA, CMI, and screening out the group of adults older than 20 years, only 3,912 individuals remained after removing individuals with outlier CMI values using the Z-Score method (**Figure 1**).

### 2.2 Definition of OSA

Drawing from prior research (24), we employed the Sleep Questionnaire (SLQ) included in the NHANES dataset to evaluate the prevalence of OSA. A diagnosis of OSA is made when an individual meets any of the following criteria: (1) experiences significant daytime sleepiness despite obtaining approximately 7 or more hours of sleep on weekdays or weeknights, occurring more than 16 times per month; (2) exhibits apnea or snorting at least three nights per week; or (3) demonstrates loud snoring at least three nights per week.

### 2.3 Definition of CMI

The CMI is calculated as follows:


$$CMI=\frac{\text{Triglycerides }(\text{mg}/\text{dL})}{\text{High-Density Lipoprotein }(\text{mg}/\text{dL})}\times \text{Waist-to-Height Ratio}$$

The triglyceride and HDL data were measured by Cobas 6000 Chemistry Analyzer.

### 2.4 Covariates

Drawing from existing literature (25–27), we incorporated several potential confounding variables as covariates in our analysis. Among these, age, poverty-to-income ratio (PIR), and total cholesterol were treated as continuous variables. Conversely, gender, race, and education level (classified as less than high school, high school, and more than high school) were considered categorical variables. Additionally, marital status (married vs. never married), smoking status (categorized based on whether the individual had smoked 100 cigarettes in their lifetime), excessive drinking status (categorized according to whether the individual consumed more than four drinks per day), as well as the prevalence of diabetes mellitus and hypertension (categorized according to whether or not they have been informed of the disease by a doctor) were also included as categorical variables.

It is important to note that due to the high correlation of variables such as BMI, height, waist circumference, high-density lipoprotein, and triglycerides with CMI, we did not consider them as associated covariates to avoid potential multicollinearity in the experimental results.

### 2.5 Statistical analysis

The statistical analysis for this study was conducted using R version 4.4.1. All statistical evaluations were weighted in accordance with the National Center for Health Statistics (NCHS) guidelines. To address the issue of missing data for additional variables (such as PIR, hypertension, diabetes mellitus, etc.) among the final cohort of 3,912 participants, we utilized the “missForest” package to perform multiple imputations, thereby minimizing the potential for multicollinearity during the analysis.

Initially, the normality of all continuous variables was assessed, revealing that they all followed a non-normal distribution. Participants were then divided into two groups according to the presence of OSA. Continuous variables were assessed using the Mann-Whitney U test, while categorical variables were analyzed with the Chi-square test. Relevant continuous variables are presented as median ± interquartile range (IQR), and categorical variables are reported as percentages (%).

In the multiple logistic regression analysis, three models were constructed: the crude model, which included no covariates; Model II, which incorporated demographic-related variables; and Model III, which included all relevant covariates. RCS regression was used to explore the nonlinear association between the CMI and OSA, while threshold effect analysis was applied to pinpoint potential inflection points in this relationship. Subgroup analyses were carried out based on factors such as age, sex, race, education level, marital status, smoking behaviors, alcohol intake, hypertension, and diabetes mellitus. Furthermore, mediation effect analysis was conducted to evaluate the mediating influence of smoking.

## 3 Results

### 3.1 Population baseline characteristics

A total of 3,912 participants were included in this study, which represents an estimated population of 90,582,166 following the application of inverse weighting. Among these individuals, 1,997 were diagnosed with OSA, resulting in a prevalence rate of 51%. The participants were categorized into two groups based on their OSA status (refer to **Table 1**). Our analysis revealed that a higher proportion of men, married people, smokers, excessive drinkers and hypertensive people had OSA compared to the other groups in the subgroups. Furthermore, those with OSA were characterized by advanced age, increased body weight, higher BMI, greater waist circumference, elevated triglyceride levels, and a higher CMI, in addition to lower values of the PIR associated with high-density lipoprotein.

**Table 1:** Weighted population baseline table.

Characteristic	Overall N = 90,582,166	Non-OSA N = 46,233,729	OSA N = 44,348,437	p-value
Age (year)	47.88± 16.83	45.68 ± 17.52	50.18 ± 15.75	<0.001
Gender (%)				<0.001
Male	48.52	44.69	52.99	
Female	51.48	55.31	47.01	
Race (%)				0.446
Mexican American	15.26	7.99	9.57	
Other Hispanic	11.94	6.70	6.52	
Non-Hispanic White	33.18	64.41	63.14	
Non-Hispanic Black	21.37	10.34	10.85	
Other Race	18.25	10.55	9.91	
Education level (%)				<0.001
Less than high school	9.10	3.62	4.96	
High school	11.71	6.60	9.29	
Above high school	79.19	89.78	85.74	
Marital status (%)				<0.001
Previously married	72.67	69.18	76.89	
Never married	27.33	30.82	23.11	
PIR	3.04± 1.58	3.10 ± 1.59	2.98 ± 1.57	0.014
Smoked at least 100 cigarettes in life(%)				<0.001
Yes	42.18	39.91	48.00	
No	57.82	60.09	52.00	
Ever have 4/5 or more drinks every day (%)				<0.001
Yes	13.50	11.51	16.31	
No	86.50	88.49	83.69	
High blood pressure (%)				<0.001
Yes	35.92	26.74	36.42	
No	64.08	73.26	63.58	
Diabetes mellitus (%)				<0.001
Yes	15.13	8.43	13.08	
No	84.87	91.57	86.92	
BMI (kg/m^2^)	29.34± 7.00	27.60 ± 6.24	31.16 ± 7.28	<0.001
Weight (kg)	83.49± 22.04	78.12 ± 19.87	89.09 ± 22.77	<0.001
Height (cm)	168.43± 9.71	167.97 ± 9.63	168.91 ± 9.76	0.003
Waist circumference (cm)	100.40± 17.09	95.79 ± 15.83	105.20 ± 17.02	<0.001
Total cholesterol (mg/dl)	189.49± 41.34	188.25 ± 41.77	190.78 ± 40.85	0.056
Triglyceride (mg/dl)	108.90± 66.82	100.31 ± 61.69	117.85 ± 70.66	<0.001
HDL (mg/dl)	55.48± 17.38	57.95 ± 18.64	52.90 ± 15.54	<0.001
CMI	1.43± 1.25	1.21 ± 1.07	1.67 ± 1.38	<0.001

Data were median (IQR) for skewed variables or proportions for categorical variables.

OSA, obstructive sleep apnea; BMI, body mass index; PIR, poverty income ratio; IQR Interquartile range; HDL, high density lipoprotein; CMI, cardiometabolic index.

### 3.2 Association between CMI and OSA

**Table 2** displays the results of the multivariate logistic regression analysis that investigates the association between CMI and OSA. We developed three separate models for this analysis, and the results consistently revealed a significant positive association between CMI and OSA across all models (p < 0.001). Specifically, in the fully adjusted model (Model III), the odds ratio (OR) was found to be 1.31, indicating that for each one-unit increase in CMI, the likelihood of developing OSA increased by 31%. Furthermore, the results of the trend analysis in the fully adjusted model indicated that individuals in the highest tertile of CMI exhibited a progressively higher incidence of OSA compared to those in the lowest tertile (p for trend < 0.001).

**Table 2:** Positive correlation between CMI and OSA.

Characteristic	Model 1 OR (95% CI)^1^, *P*-value	Model 2 OR (95% CI)^1^, *P*-value	Model 3 OR (95% CI)^1^, *P*-value
CMI (continuous)	1.37 (1.26, 1.49) <0.001	1.32 (1.21, 1.43) <0.001	1.31 (1.21, 1.42) <0.001
CMI (categorization)			
Quartile1	Reference	Reference	Reference
Quartile2	1.68 (1.31, 2.15) <0.001	1.55 (1.20, 1.99) 0.002	1.54 (1.19, 2.00) 0.003
Quartile3	2.66 (2.11, 3.35) <0.001	2.33 (1.83, 2.98) <0.001	2.28 (1.81, 2.87) <0.001
*P* for trend	<0.001	<0.001	<0.001

*^1^* OR, Odds Ratio, CI, Confidence Interval.

Model 1: No covariable adjustment.

Model 2: Age, gender, race, education, marital status and PIR adjustments.

Model 3: Age, gender, race, education, marital status, PIR, total cholesterol, smoking status, hypertension, diabetes mellitus and alcohol consumption status were all adjusted for.

### 3.3 Restricted cubic spline analysis with threshold effect analysis

**Figure 2:**
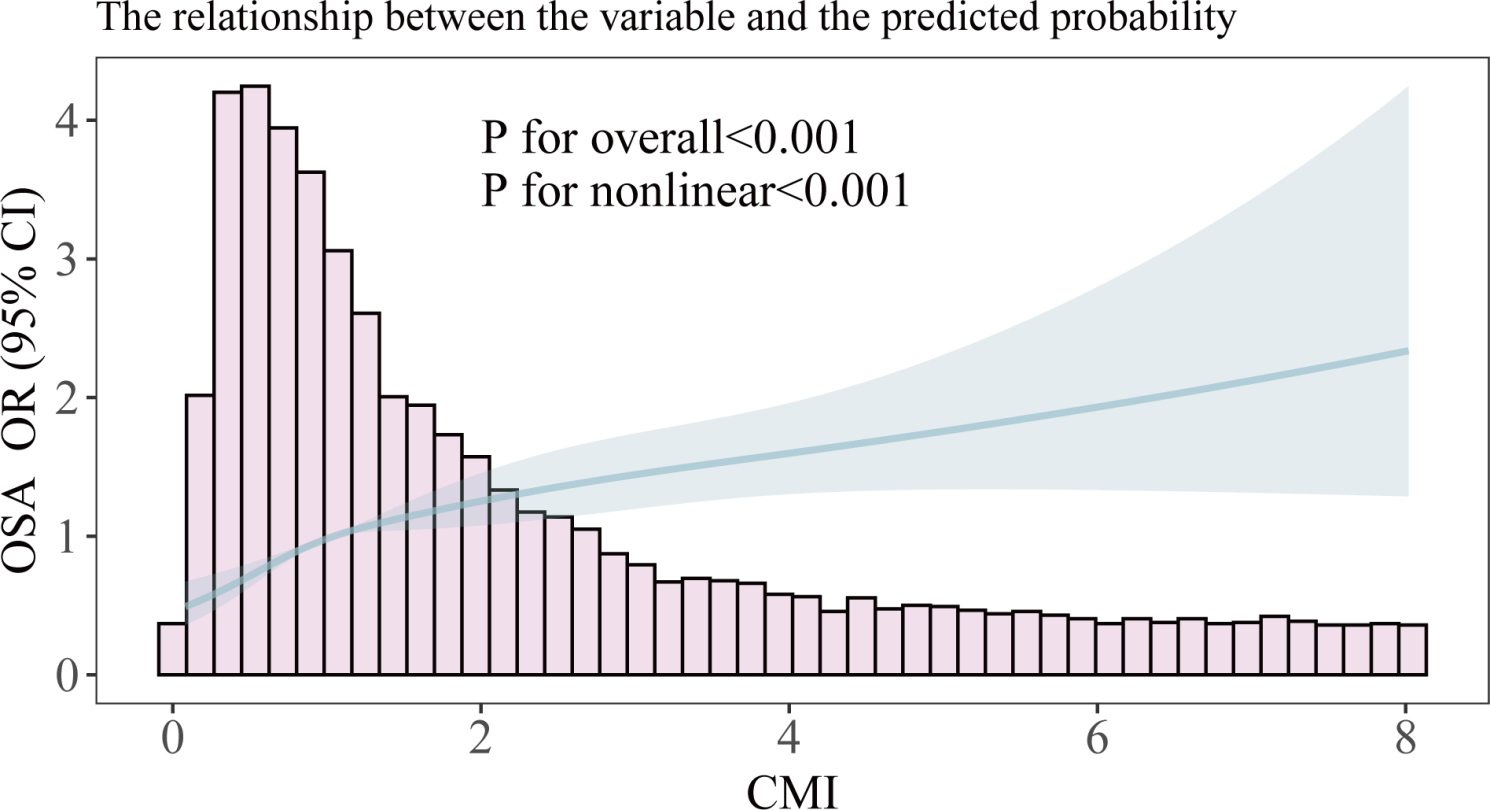
Smooth curve fitting of association between CMI and OSA. The solid portion and the shaded portion represent the predicted values and 95% confidence intervals, respectively. The model is model 3 after adjusting for all relevant covariates.

We employed restricted cubic splines to analyze the nonlinear relationship between the CMI and OSA (**Figure 2**). The results indicated a positive correlation between CMI and OSA in the fully adjusted model (Model III), with a statistically significant overall p-value of less than 0.001. This finding reinforces the earlier conclusions drawn from the multiple logistic regression analysis and suggests a notable nonlinear relationship between these two variables (p for nonlinear < 0.001). We observed what appeared to be a “L” shape relationship, prompting us to conduct a threshold effect analysis, which identified a significant inflection point at 0.582 (p for likelihood ratio test < 0.001). Prior to reaching this inflection point, the prevalence of OSA was found to increase by a factor of 4.89 for each one-unit rise in CMI; conversely, beyond this point, the prevalence increased by only 0.19 for each unit increment in CMI (refer to **Table 3**).

**Table 3:** Threshold effect analysis of CMI and OSA.

OSA	The effect size, (95% CI)	*P*-value
CMI		
Model I	1.25(1.18-1.33)	<0.001
Model II		
Inflection point	0.582	
< 0.582	5.89(2.93-11.95)	<0.001
> 0.582	1.19(1.11-1.26)	<0.001
P for likelihood ratio test		<0.001

Age, gender, race, education, marital status, PIR, total cholesterol, smoking status, hypertension, diabetes mellitus and alcohol consumption status were all adjusted for.

Model I: Fitting model by standard linear regression.

Model II: Fitting model by two-piecewise linear regression.

### 3.4 Subgroup analysis

**Figure 3:**
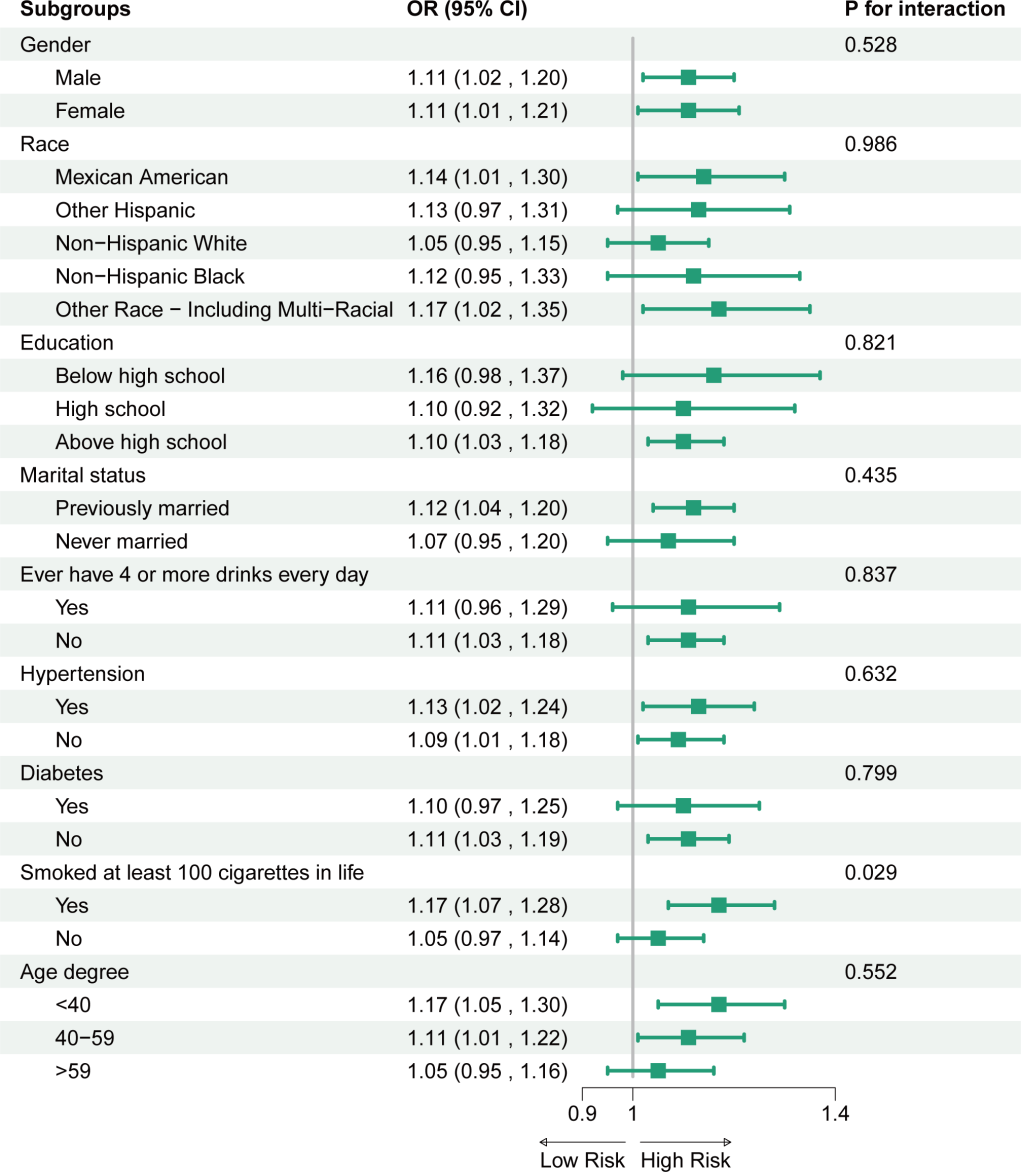
Association between CMI and OSA. For each stratification element we used Model 3 (fully adjusted model) for the adjustment analysis, except for the stratificationfactors themselves.

To further investigate the stratified association between CMI and OSA within subgroups, we analyzed participants based on various demographic characteristics, as well as smoking status, excessive alcohol consumption, prevalence of hypertension, and prevalence of diabetes mellitus (**Figure 3**). The results showed that the positive correlation between CMI and OSA was broadly present across subgroups. Notably, we identified a significant interaction effect based on smoking status (p for interaction < 0.05), revealing that smokers exhibited a stronger positive association between CMI and OSA compared to non-smokers.

### 3.5 Mediation effect analysis

**Figure 4:**
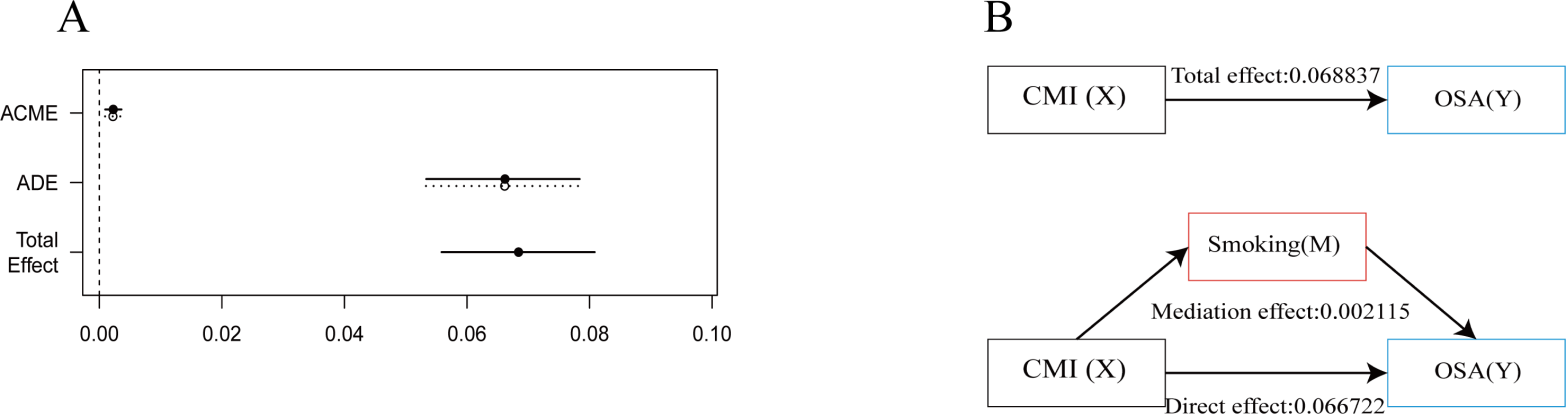
Analysis of intermediation effects. This figure shows the mediation model of the independent variable CMI, smoking as the mediating variable, and the dependent variable OSA. **(A)** Effect Values and Confidence Intervals for the CMI, OSA and smoking Mediating Effects (ACME, average causal mediation effects; ADE, Average Direct Effect). **(B)** Path diagram of mediation analysis of the relationship between smoking on CMI and OSA.

The results from the interaction analysis indicated that individuals who smoke have a more pronounced positive correlation between their CMI and OSA when compared to non-smokers. Consequently, we further investigated the potential mediating effect of smoking in this relationship. The mediating effect of smoking is illustrated in **Figure 4**, with additional data available in the **Supplementary Material**. The findings demonstrated that the total effect of CMI on OSA is 0.068837. After adding smoking as an intermediate medium, the direct effect of CMI on OSA is 0.066722. Smoking partially mediated the association between CMI and OSA, yielding a mediating effect size of 0.002115 (p < 0.001). This implies that smoking serves as a positive intermediary factor influencing the relationship between CMI and OSA.

## 4 Discussion

The findings of this study reveal a significant positive nonlinear relationship between elevated CMI and the incidence of OSA in the adult population of the United States. The “L” shape relationship found through threshold effect analysis indicates that maintaining an appropriate CMI level is critical to mitigating the risk of developing OSA. Specifically, our analysis demonstrated that for each unit increase in CMI, the likelihood of OSA increases by 31% in the fully adjusted model [OR (95% CI): 1.31 (1.21, 1.42), p < 0.001]. Notably, smoking was found to mediate the association between CMI and OSA, highlighting its role as a confounding factor that exacerbates this risk.

In practical terms, these results suggest that CMI could be effectively integrated into existing risk stratification frameworks for OSA screening. Healthcare providers can utilize CMI as a quantifiable metric to identify individuals at heightened risk for OSA, allowing for timely interventions. By incorporating CMI into routine assessments, clinicians could enhance their ability to stratify patients based on risk and customize preventive strategies tailored to individuals’ profiles. For example, patients with high CMI scores could undergo more rigorous screening for OSA, enabling earlier diagnosis and targeted management, potentially including lifestyle modifications or therapeutic interventions. Overall, adopting CMI in clinical practice as part of risk stratification for OSA could significantly improve patient outcomes and empower healthcare providers to implement proactive measures against this common sleep disorder.

Obstructive Sleep Apnea (OSA) is increasingly acknowledged as an important public health concern due to its high prevalence and serious health consequences. Research estimates that about 25% of adult males and 10% of adult females in the United States are impacted by OSA (28, 29), with these rates continuing to rise alongside increasing obesity rates (30). This sleep disorder is marked by repeated instances of either complete or partial blockage of the upper airway during sleep, which results in intermittent hypoxia and frequent awakenings. The consequences of untreated OSA are significant, leading to various adverse health outcomes, such as cardiovascular disease, hypertension, type 2 diabetes, and an increased risk of stroke (31–36). Additionally, OSA is associated with decreased quality of life, increased healthcare utilization, and economic burdens, underscoring the urgent need for effective screening and intervention strategies.

The cardiometabolic Index (CMI) has emerged as a valuable tool for assessing cardiovascular and metabolic health. It integrates multiple physiological parameters, such as waist-to-height ratio (used to reflect abdominal obesity), and lipid levels (used to reflect lipid metabolism in the body), to provide a comprehensive evaluation of an individual’s cardiac health (37–39). Its significance lies in its potential to identify individuals at increased risk for cardiovascular complications, thereby enabling proactive management of metabolic syndrome (15, 16, 40, 41). The development of CMI reflects a growing awareness of the interactions between metabolic health and cardiovascular disease, highlighting the importance of adopting a holistic approach to patient care.

In this study, we identified a significant positive correlation between the CMI and OSA. This finding suggests that cardiac metabolic health may play an important role in the occurrence and progression of OSA. Several potential mechanisms could explain this correlation. First, CMI is derived from a combination of factors such as waist circumference, height, triglycerides, and high-density lipoprotein levels, which collectively reflect an individual’s metabolic status and fat distribution. A higher CMI is typically indicative of abdominal obesity and metabolic dysfunction (14, 42). These factors can lead to fat deposition in the upper airway tissues (18, 43), increasing airway resistance and contributing to the development of OSA. Additionally, abdominal obesity may impair diaphragm function (44), further disrupting normal nighttime respiration. Second, elevated CMI is often closely linked with the development of insulin resistance, chronic inflammation, and metabolic syndrome (15, 45–48). Insulin resistance can lead to abnormal adipocyte function and the production of pro-inflammatory mediators (49–52), which not only affect cardiac metabolic health but may also exacerbate OSA by interfering with neural control of respiration (53, 54). Researches have shown that chronic inflammation is associated with upper airway dysfunction and dysregulation of neural control, both of which can worsen OSA (20). Moreover, the deterioration of cardiovascular health may also exacerbate the symptoms of OSA by affecting the balance of the autonomic nervous system. An increase in CMI is often accompanied by elevated blood pressure and greater cardiac workload, changes that may interfere with the neural regulation of breathing (55, 56), resulting in increased frequency of nocturnal apneas. Recent studies have shown that Glucagon-like peptide-1 receptor agonists (GLP-1RAs) and sodium-glucose cotransporter-2 (SGLT2) inhibitors have emerged as promising candidates. Both classes of medications, initially designed for T2D and obesity management, offer pleiotropic cardiovascular benefits, including weight reduction, blood pressure lowering, and improvements in endothelial function. Clinical studies suggest that GLP-1RAs can decrease OSA severity and enhance daytime alertness by mitigating upper airway fat accumulation and enhancing respiratory control. Similarly, SGLT2 inhibitors may exert beneficial cardiovascular effects through their actions on myocardial energetics and renal function (57–59). These mechanisms provide further insight into the complex pathology of OSA and emphasize the need for consideration of cardiac metabolic health in the clinical management of this condition. Future research should explore these mechanisms in greater depth to offer new strategies for the early identification and intervention of OSA.

Additionally, we found that smoking serves as a mediating factor, exacerbating the positive correlation between CMI and OSA. This may be attributed to the fact that smoking not only induces metabolic disturbances but also compromises respiratory health, thereby elevating the risk of OSA in individuals with high CMI (60–62). These results highlight the significance of focusing on metabolic health and lifestyle factors, such as smoking cessation, in the strategies for preventing and managing OSA (63).

## 5 Advantages and limitations

We believe that this study, which specifically investigates the relationship between the CMI and OSA, presents a novel and significant contribution to the field. Throughout the research, we employed appropriate statistical methods and ensured continuity and logical flow by using the results from previous analyses to inform subsequent ones. The findings from the threshold effect and mediation effect analyses also offer valuable insights for clinical practice and daily life. However, since this research adopts a cross-sectional study design, the data collection is focused on a specific point in time, thereby limiting the ability to establish causal relationships among the variables. The nature of cross-sectional research prevents the identification of longitudinal trends and potential temporal relationships, which are essential for understanding the dynamics of the factors examined. Therefore, the limitations of this specific dataset hinder our ability to infer causal relationships between CMI and OSA, and to ascertain the causal role of smoking in the mediating effects between the two. Second, the constraints of covariates (key cardiovascular metabolic drugs, especially newer drugs such as GLP-1 receptor agonists and SGLT2 inhibitors), may simultaneously affect CMI and OSA. Unfortunately, due to the lack of data on the use of these drugs in the NHANES database, we were unable to include them as covariates in this study. Third, in our study, the diagnosis of OSA primarily relied on self-reported symptoms evaluated through the Sleep Questionnaire within the NHANES dataset. While this approach provides valuable insights into the prevalence of OSA, it inherently lacks the rigor of objective diagnostic methods, such as polysomnography (PSG) or assessing apnea-hypopnea index (AHI) scores. The reliance on subjective reporting may result in misclassification bias, as individuals may overreport or underreport their symptoms, leading to an inaccurate assessment of OSA prevalence. Consequently, this limitation underscores the need for a multidimensional evaluation that integrates both subjective symptom assessment and objective diagnostic criteria. Future investigations should aim to incorporate objective measures alongside self-reported questionnaires to enhance the accuracy of OSA diagnosis and to better understand its implications for public health and clinical practice. In addition, potential biases from self-reported questionnaires, including recall bias and loss to follow-up, may have affected the results. Finally, it is important to note that the study sample consists exclusively of American adults, which may restrict the generalizability of the results to other populations. Variations in culture, socioeconomic status, and behavior across different demographic groups could affect the study variables, suggesting that the findings may not be relevant to individuals from outside this demographic. Future studies should further investigate longitudinal effects and related intervention strategies to improve our comprehension of the mechanisms that connect CMI to OSA and to refine the experimental findings.

## 6 Conclusion

Our study reveals a positive nonlinear relationship between the CMI and OSA in the adult U.S. population. Threshold analysis indicates that keeping the CMI within a specific range may significantly lower the likelihood of developing OSA. Additionally, lifestyle factors like smoking may further influence this relationship. These findings highlight the importance of a comprehensive approach to screening and intervention that considers cardiometabolic and sleep health. Future research should focus on understanding the mechanisms linking CMI and OSA to improve prevention and treatment strategies for at-risk individuals.

## Data Availability

The original contributions presented in the study are included in the article/**Supplementary Material**. Further inquiries can be directed to the corresponding author.
