# Supplementary figures and images for: Association between cardiometabolic Index and obstructive sleep apnea and the mediating role of smoking: a cross-sectional study
Source: Front Endocrinol (Lausanne). 2025 Jul 9;16:1609585. doi: 10.3389/fendo.2025.1609585 (PMC12283312; doi:10.3389/fendo.2025.1609585)

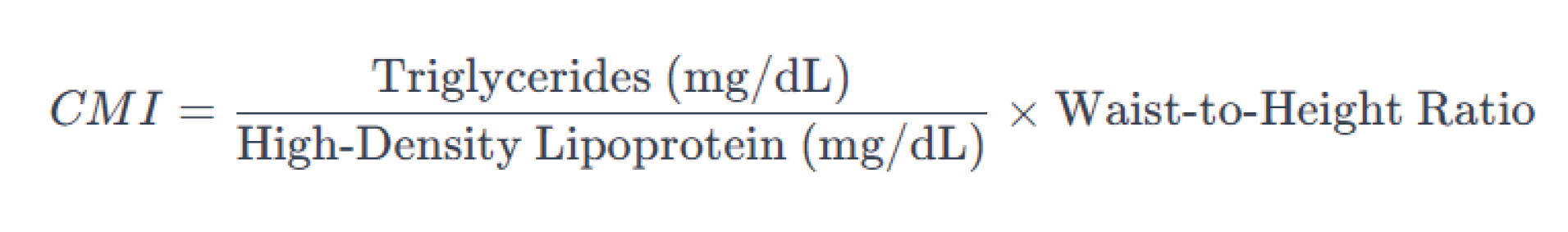

Supplement: Supplementary file 1 [file Image1.tif]
