# Supplementary material for: Association between cardiometabolic Index and obstructive sleep apnea and the mediating role of smoking: a cross-sectional study
Source: Front Endocrinol (Lausanne). 2025 Jul 9;16:1609585. doi: 10.3389/fendo.2025.1609585 (PMC12283312; doi:10.3389/fendo.2025.1609585)
Supplement: Supplementary file 2 [file Table1.docx]

**Supplementary Table.S1.**

**Smoking as a mediator of the association between the CMI and OSA.**

| **Mediation effect** | **Estimate** | **95 % CI lower** | **95 % CI upper** | ***P*-value** |
| --- | --- | --- | --- | --- |
| Total effect | 0.068837 | 0.057761 | 0.08 | ＜0.0001 |
| Mediation effect | 0.002115 | 0.000845 | 0.003 | ＜0.0001 |
| Direct effect | 0.066722 | 0.055459 | 0.08 | ＜0.0001 |
| Proportion mediated | 0.029233 | 0.011679 | 0.06 | ＜0.0001 |
